# Exploring music preferences, behaviours and experiences of exercising to music in pulmonary rehabilitation for individuals with chronic respiratory diseases: a cross-sectional survey

**DOI:** 10.1136/bmjoq-2025-003666

**Published:** 2026-01-12

**Authors:** Omar A Alhothaly, Linzy Houchen-Wolloff, Sarah Ward, Emma Chaplin, Jakub Zatloukal, Mark Dunlop, Sally J Singh, Mark W Orme

**Affiliations:** 1 University of Leicester Department of Respiratory Sciences, Leicester, UK; 2 Centre of Exercise and Rehabilitation Sciences, NIHR Leicester Biomedical Research Centre - Respiratory, University Hospitals of Leicester, Leicester, UK; 3 University of Strathclyde Department of Computer and Information Sciences, Glasgow, UK

**Keywords:** Rehabilitation, Patient Preference, Quality improvement, Exercise

## Abstract

**Background:**

Music can enhance exercise performance, but its potential has not been well explored in pulmonary rehabilitation (PR). The aim was to explore the current music-related behaviours among PR service users with chronic respiratory diseases (CRDs) to inform future PR service interventions and explore the potential for music to facilitate exercise adherence in this context.

**Methods:**

The cross-sectional survey was distributed among PR attendees at the University Hospitals of Leicester (UHL) NHS Trust in the United Kingdom, between November 2023 and August 2024. Participants completed a 25-item survey exploring (i) relevant technology ownership and music-related behaviours, (ii) preferred music genres and songs and (iii) anticipated benefits/concerns of exercising to music. Quantitative data were analysed descriptively. Free-text data were analysed using qualitative counting.

**Results:**

We surveyed 109 people living with CRDs (51% male, 56% aged ≥70 year, 76% chronic obstructive pulmonary disease, 82% owned a smartphone). More than half had no prior experience of exercising to music (n=59, 54%). Despite this, almost half of participants listened to music at least once/day (n=54, 49%), primarily via the radio (n=83, 76%) and/or online music platforms (n=76, 70%). Pop (n=39, 36%) and Country (n=38, 35%) were the most popular music genres listened to, with the majority listening to music without headphones (n=64, 59%). The main concern about wearing headphones while exercising was that it might reduce their awareness of the surroundings (n=67, 61%). The perceived benefits of listening to music during exercise were to boost their mood (n=39, 36%) or help maintain their walking pace (n=19, 17%).

**Conclusion:**

There is potential to use music as a tool to support exercise in PR. However, lack of prior experience exercising to music, diverse music preferences, safety considerations and the need to increase knowledge of the potential benefits of exercising to music are key challenges. These findings may help future PR services to implement music into their programmes and develop personalised music-based interventions to optimise exercise performance.

**WHAT IS ALREADY KNOWN ON THIS TOPIC**

- Music listening can reduce dyspnoea, fatigue and perceived exertion in people with chronic respiratory disease, but self-selected music use is uncommon in pulmonary rehabilitation (PR), and little is known about PR attendees’ everyday music behaviours or preferences.

**WHAT THIS STUDY ADDS**

- This survey identifies a high prevalence of music listening in daily life but limited experience of exercising to music, with reports of safety concerns about outdoor headphone use.
- The findings highlight a high diversity in genre and artist preferences, emphasising the need for personalised approaches rather than generic playlists.

**HOW THIS STUDY MIGHT AFFECT RESEARCH, PRACTICE OR POLICY**

- Understanding PR attendees’ music behaviours can guide the design of personalised music-based interventions to support exercise adherence in a safe and acceptable manner.
- The survey identifies key implementation barriers, including perceived concerns, lack of prior experience with exercising to music and high diversity of music preferences that future interventions should consider.

## Introduction

 Pulmonary rehabilitation (PR) is an evidence-based intervention known to improve physiological and psychological symptoms for patients with chronic respiratory disease (CRD).^1^,^3^ Exercise training, particularly walking, is a core component of PR.^2 3^ Chronic cough, fatigue and breathlessness are common symptoms of CRDs which limit people’s confidence and capability to participate in walking and other forms of daily activities and exercise.^4^ Factors such as perception of breathlessness and fatigue, lack of motivation and social support, reduced exercise tolerance and further impact the long-term adherence to unsupervised home-based exercise, including walking.^3^ Previous research has shown that even with using tools (eg, commercial activity monitor) which might increase the walking exercise participation in people with chronic obstructive pulmonary disease (COPD) attending PR, 50% of the exercise steps were not taken at the individually prescribed walking intensity.^5^

Music has shown potential to reduce perception of dyspnoea^6^ and fatigue,^7^ as well as increase enjoyment^8^ and support greater exercise engagement in people with CRDs. These potential benefits have led music to be used as an intervention.^6 9^ Auditory feedback in the form of listening to music offers a valuable tool during exercise for healthy individuals^10 11^ and for people living with COPD, serving as a positive distraction from fatigue and dyspnoea to enhance performance.^712^,^15^ For example, listening to music as a distractive auditory stimulus while walking for exercise following PR (maintenance programme) improved functional performance and reduced the perception of dyspnoea compared with exercising without music.^6^

Researchers have explored exercising to the tempo of the music as measured in beats per minute (bpm) to support exercise adherence.^1116^,^18^ For example, exercising to fast music (90–120 bpm) may improve exercise performance in people with COPD.^6 13 16 19 20^ Synchronising a person’s step count while walking, with the tempo of music being listened to, has been shown to be effective in improving functional performance, reducing the perceptions of breathlessness and fatigue and improving health-related quality of life for people with CRD.^16^,^18^ This approach has been suggested as an effective tool to increase the adherence to walking for exercise by offering a more enjoyable and engaging exercise experience.^16^ Personalised music-based interventions remain underutilised in PR, with a recent survey of clinicians in Australian PR centres reporting that listening to self-selected music for individual use is uncommon for participants during PR.^21^ It is unknown what PR attendees’ music-related behaviours are (eg, how, where and when participants listen to music), the barriers and facilitators of using music-based interventions in the context of PR.^22^ Exploring PR attendees’ music preferences and behaviours is needed to facilitate the development of personally tailored music-based interventions for PR services. Accordingly, the aim of this survey was to explore the current music-related behaviours among PR service users with CRD to inform future PR service interventions and explore the potential for music to facilitate exercise adherence in PR.

## Methods

### Survey design

A cross-sectional survey was conducted and registered as a service-improvement project (Reference: 12626) at the University Hospitals of Leicester (UHL) NHS Trust in the United Kingdom between November 2023 and August 2024 and therefore, was exempted from ethical approval. This report was prepared in adherence to the Strengthening the Reporting of Observational Studies in Epidemiology (STROBE) checklist for cross-sectional studies.^23^

### Procedure

The survey was available in paper and digitally on the Joint Information Systems Committee online surveys platform (JISC Services Ltd, Bristol, UK) and complied with the General Data Protection Regulation requirements.^24^ After verbal consent, participants completed the survey using a paper-based or online version. Data were collected directly from participants during PR classes or appointments or during telephone calls as part of their programme. The surveys took approximately 10–15 min to complete, and all responses were anonymised.

### Survey description

The survey included 25 closed or open-ended questions (online supplemental material A). The survey questions explored three themes: (i) relevant technology ownership and music-related behaviours, (ii) music listening experiences, including preferences and perception of benefits and concerns of listening to music while exercising, and (iii) patient demographics.

### Patient and public involvement

The survey was piloted with PR service users for feedback and amendments on content, clarity and ease of use. At the survey design stage, copies of the survey were piloted with PR service users at the time of their usual PR classes. Examples of changes made include the following: (i) brief definitions and illustrative images were added before questions related to smartphone and headphones or earbuds to help participants better understand the context; (ii) some questions were modified from single-answer format (selecting only one response) to multiple-choice format (select all that apply); and (iii) additional response categories were included based on the PR service users’ feedback. For example, ‘Alexa’ was added to the answer options for questions related to modes/ways of listening to music.

### Participants

Given the exploratory nature of this survey, the aim was to gather baseline knowledge, insights and opinions on PR attendees’ music and technology usage to inform PR service delivery. As such, a convenience sample of individuals referred to and/or enrolled in PR at the (Site) during the study period was approached to take part. No predefined target sample size was set; recruitment continued until the end of the data collection period. Participants were eligible for the survey if they met the PR eligibility criteria outlined in the British Thoracic Society guidelines^25^ and were taking part in PR at (Site).

### Bias

To minimise selection bias, efforts were made to provide a translation for individuals who could not write and/or understand written or spoken English language. Where possible, a volunteer of the clinical team who spoke the language explained the purpose of the survey. Participants who could not read or write English were also asked if they had a family member who could help with completing the survey.

### Statistical methods

All data were entered manually (for paper-based responses) or digitally (for online responses) into the JISC online survey platform. To ensure data quality, all manual entries were double-checked by the researcher against the source document (paper-based survey) for errors, inconsistencies and missingness. Any inconsistencies identified during the process were reviewed and resolved (where possible) before analysis. Once data were entered, responses were exported to a Microsoft Excel Worksheet for analysis. Respondents were considered completers if they answered at least 80% (20 questions) of the survey.^26^

All quantitative data (categorical and ordinal) were analysed using descriptive statistics using frequency and percentage. Free-text questions were analysed using qualitative counting.^27^ Specific words or phrases were categorised into different groups, and their frequency of use in response to a particular question was recorded and summarised.

## Results

### Participants

A total of 121 participants were approached to take part in the survey. Out of these, 110 agreed to participate and 109 provided sufficient data for analysis (table 1). Three participants required translation (all into Urdu) to complete the survey, and one participant had support with completing the survey from a family member.

**Table 1:** Demographic and clinical characteristics

Characteristics	Participants, n (%)
Gender	
Male	56 (51)
Female	53 (49)
Age category (years)	
<50 years	1 (1)
50–59 years	13 (12)
60–69 years	33 (30)
70–79 years	45 (41)
≥80 years	17 (15)
Chronic respiratory diseases*	
Chronic obstructive pulmonary disease	83 (75)
Asthma	25 (23)
Bronchiectasis	17 (15)
Interstitial lung disease	10 (9)
Other†	8 (7)

* The total percentage exceeds 100%, as some participants had multiple diagnoses.

† Pulmonary hypertension, exertional dyspnoea (undiagnosed), diaphragmatic dysfunction, pulmonary sarcoidosis, psychogenic dyspnoea, multifactorial dyspnoea, diaphragmatic palsy, lung fibrosis.

### Technology ownership and usage

Most participants owned a smartphone (n=89, 82%), of which the majority were Android (n=55, 62%). Out of those smartphone owners, the majority reported knowing how to instal a smartphone application (n=63, 71%). Over a third had prior experience using smartphone applications to monitor their health or for exercising purposes (n=35, 39%). The majority of respondents reported spending 2 hours or less per day on smartphones (n=60, 67%). Responses indicated that 41% (n=45) reported using headphones for listening to music.

### Music preferences and behaviours

#### Frequency, locations, reasons and methods of listening for different audio activities

A summary of the frequency, locations, reasons and methods of listening for different audio activities is provided in online supplemental table 1. Almost half of participants listened to music at least once/day (n=54, 49%), mainly via the radio (n=83, 76%) or online music platforms (n=76, 70%). Similarly, the most common audio content listened to daily was music radio stations (n=42, 39%) and personal music on digital devices (n=19, 18%), whereas podcasts (n=4, 4%) and audiobooks (n=2, 2%) were the least popular.

The most common location for listening to audio content was the home (eg, n=46, 42% for personal music collections from non-digital devices). Vehicles were the most common location for music radio station listening (n=73, 67%).

Respondents’ main reasons for listening to music radio stations were for background listening (n=62, 57%). Relaxation was mainly the reason for listening to personal music collections (n=51, 47%), music radio stations (n=51, 47%) and watching music videos (n=23, 21%). Respondents reported that listening to speech-based radio stations was for their interest (n=22, 20%) and to keep them updated with the news (n=19, 17%).

The majority of respondents never listened to audiobooks (n=85, 78%), podcasts (n=80, 73%) or speech-based radio stations (n=62, 57%).

#### Music listening experiences and preferences

**Figure 1:**
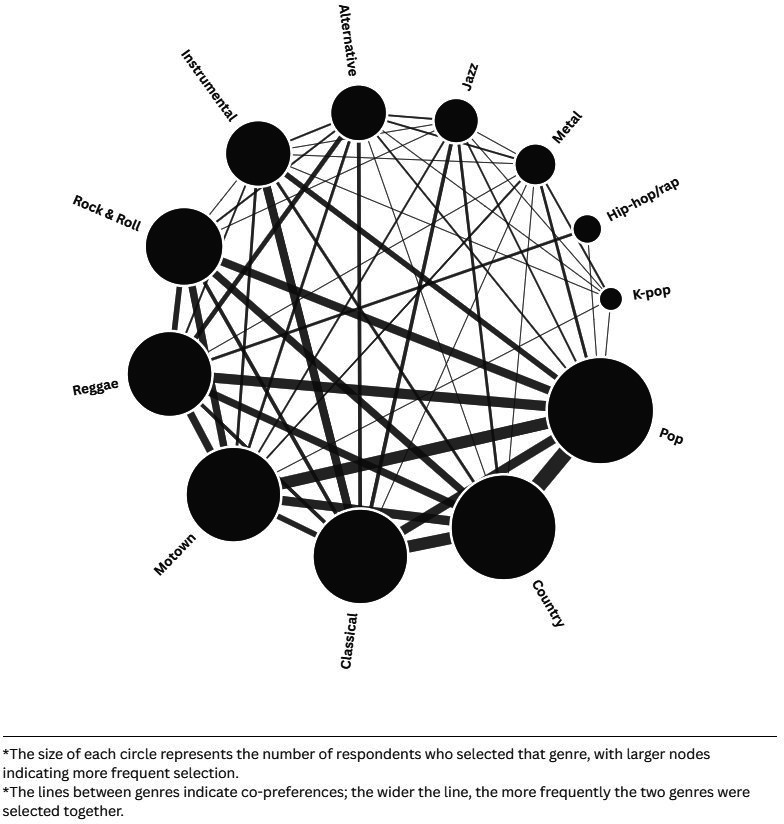
Network plot of the preferred music genre reported by respondents.

The favourite music genres selected by PR service users showed the top 3 to be Pop (n=39, 36%), Country (n=38, 35%) and Classical (n=31, 28%) (online supplemental table 2A). A total of 51 different genre pairings were selected together by respondents. The most frequently selected genre pairs were Country and Pop (n=17), Classical and Country (n=13), Pop and Motown (n=13), Pop and Reggae (n=11), and Country and Motown (n=10) (figure 1; online supplemental table 2B).

**Figure 2:**
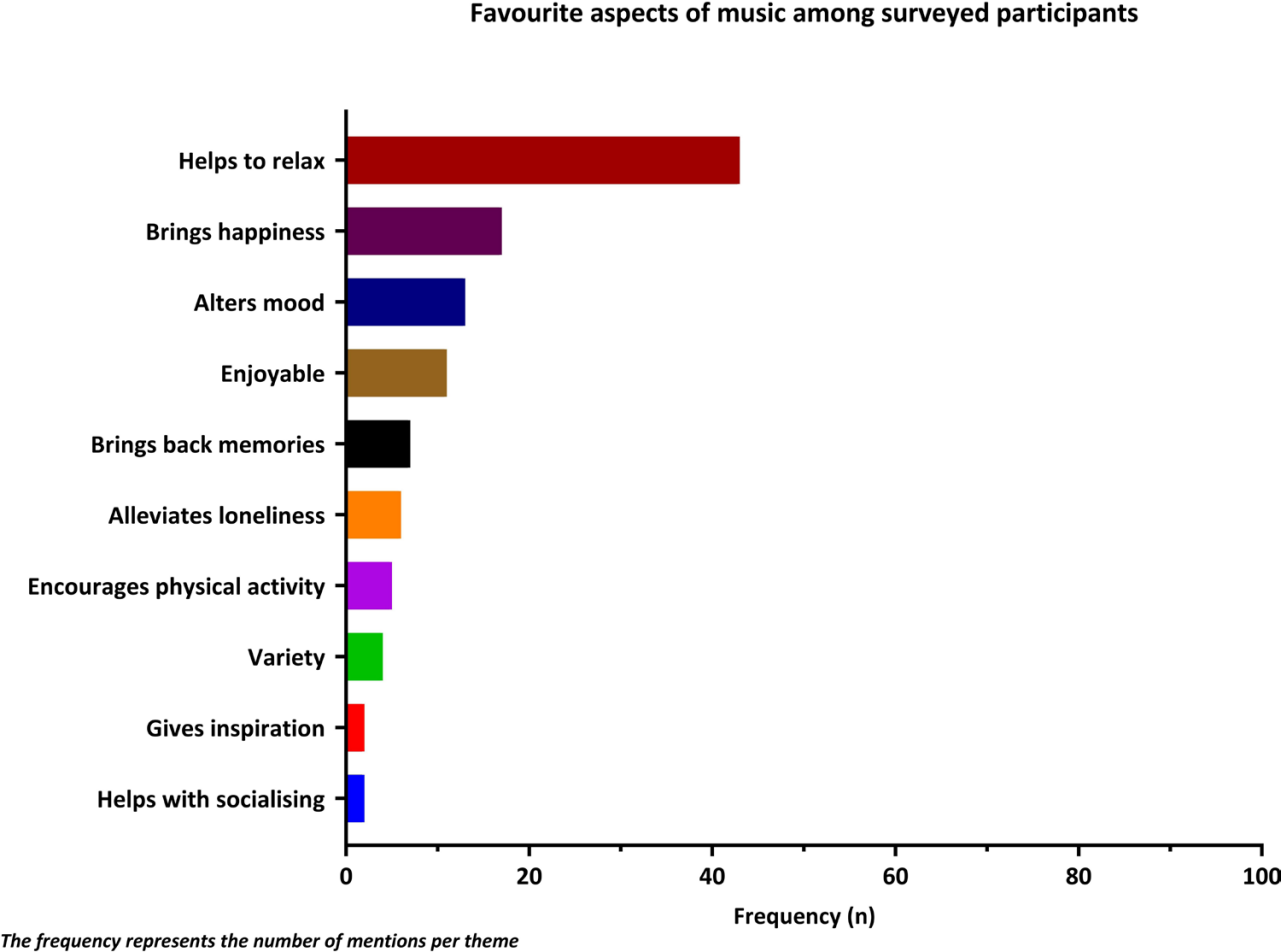
Favourite aspects of listening to music among surveyed participants.

Out of 109 respondents, 87 participants (80%) reported 181 different favourite artists/bands. The top 3 favourite artists/bands were The Beatles (n=11, 10%), Queen (n=11, 10%) and Bob Marley (n=9, 8%), (online supplemental table 3A). Similarly, 72 participants (66%) reported 199 different favourite songs, with the top 3 songs as ‘Bohemian Rhapsody’ (n=6, 8%), ‘Red Red Wine’ (n=6, 8%) and ‘No Woman No Cry’ (n=3, 4%) (online supplemental table 3B). Most participants (n=90, 83%) reported that their favourite aspects of music were helping them to relax (n=43, 39%) and bringing them happiness (n=17, 16%) (figure 2).

#### Experiences of exercising to music

**Figure 3:**
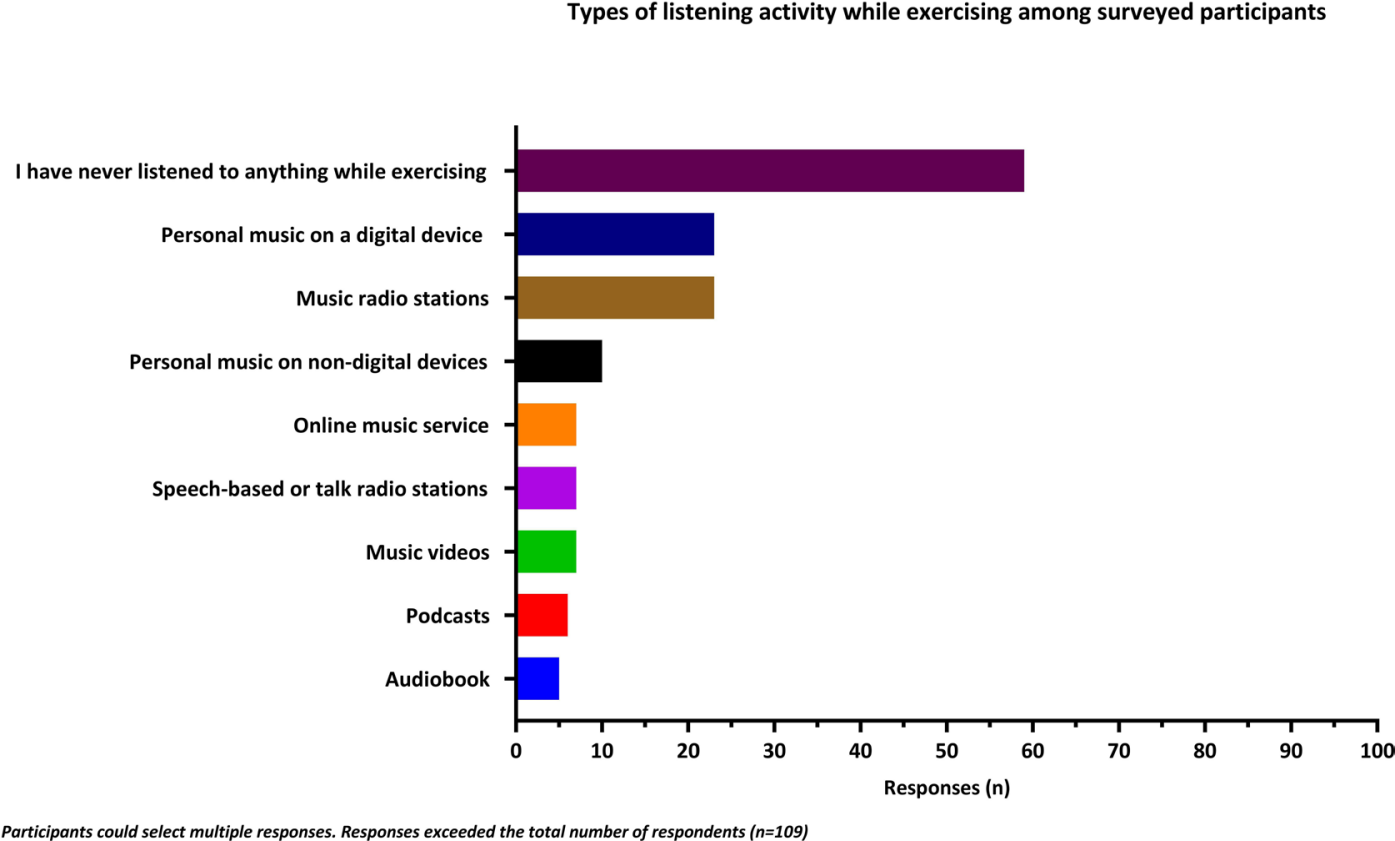
The types of listening activities participants listened to while exercising.

More than half of participants had no prior experience exercising to music (n=59, 54%). Of the participants with some experience exercising to music, the most common activities during exercise were listening to personal music collection via a digital device (eg, Smartphones, n=23, 21%) or listening to music radio stations (n=23, 21%) (figure 3).

#### Expectations and concerns of exercising to music

**Figure 4:**
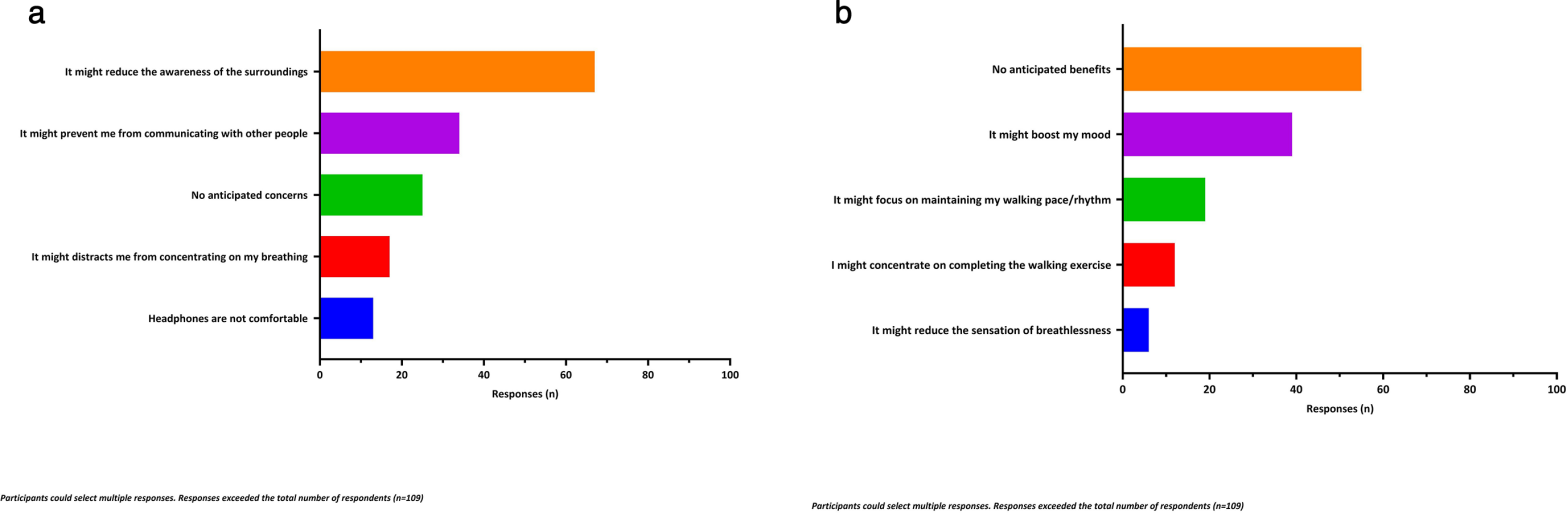
(**a**) Perceived concerns and benefits of listening to music from headphones while exercise walking outdoor. (**b**) Perceived benefits of listening to music from headphones while exercise walking outdoor.

The main concerns about wearing headphones while walking outside were that it might reduce awareness of surroundings (n=67, 61%), hinder communication with others (n=34, 31%) or distract them from concentrating on their breathing (n=19, 17%) (figure 4a). Half of the participants reported not anticipating any benefits from exercising to music (n=55, 50%), of which (n=38, 69%) had never previously exercised to music. Of the remaining responses, the most common potential benefits of listening to music during exercise were to boost their mood (n=39, 36%) or help them focus on maintaining their walking pace (n=19, 17%) (figure 4b).

## Discussion

### Main findings

This is the first survey to explore music preferences, behaviours and perceptions of exercising to music of people living with CRDs taking part in PR. Our findings showed that a high proportion of PR service users own smartphones, but few listen to music through this medium and, instead, listen to radio music more. Responses showed a diversity of music preferences. Most respondents listened to music without headphones, citing safety concerns about listening to music through headphones when walking outdoors. The majority of participants had no prior experience exercising while listening to music and did not anticipate benefits from exercising to music. These findings will provide valuable information to consider when integrating a music-based intervention into the PR, specifically when aiming to improve exercise adherence.

### Music listening is prevalent in daily use among PR attendees but not for exercise raising awareness of the potential benefits of exercising to music is needed

Listening to self-selected music during PR for individuals with COPD can enhance exercise-related motivation by making sessions more enjoyable and relaxing.^28^ However, there is limited information about utilising music listening while exercising in PR centres. Some programmes reported playing background music during classes, whereas fewer seem to be using self-selected music.^21^ However, it is unclear how many PR attendees listen to self-selected music in these reported PR programme centres. From our survey, the respondents reported frequent music listening, but not for exercising purposes, with many reporting no prior experience of exercising to music. While music appears to be an important aspect of daily life for many individuals with CRD, there may be a lack of awareness of the potential benefits of exercising to music. Incorporating the potential benefits of exercising to music into PR could improve the exercise experience of service users. Given that PR service users often find it difficult to walk for exercise at their individualised intensity,^5^ a personalised (self-selected) music experience may play a role in facilitating more effective unsupervised exercise.

### Personalising music to support exercising is needed, and diversity of the music genre/songs should be considered

Participants’ music preferences may play a key role in enhancing the positive impacts of music listening during exercise, compared with ‘one size fits all’ music playlists.^8^ Given the high diversity of preferred music genres and songs in the present survey, using music for exercising purposes should be individually tailored to optimise the psychophysiological effect of music.^29 30^ Personalising music playlists for exercising may ensure the positive affective quality of music, which includes feelings of pleasure, enjoyment or satisfaction, all of which can influence decision-making processes related to exercise adherence.^31 32^ However, individualising an exercise playlist for all patients may be unfeasible in a clinical setting. PR attendees should be encouraged to create their preferred music playlists to listen to during their individual supervised exercises (eg, treadmill, cycling) or for unsupervised home-based walking exercise. Music tempo (eg, fast tempo music ≥120 beat per minute) has been linked to influence exercise performance by increasing arousal, reducing perceived exertion^31^ and preferred music may increase motivation to exercise.^8^ However, matching both music tempo and music preference with the prescribed exercise intensity (eg, walking exercise) may be challenging. Therefore, there is a need for future interventions promoting exercise in CRD populations to explore alternatives to matching music tempo with exercise intensity.^33^ Such a tool may help service users to maintain the exercise intensity while benefiting from the positive effect of self-selected music/audio content.

### Paced walking to music tempo may be challenging given the radio popularity

Paced walking to music by synchronising walking steps to music tempo during home-based exercise for individuals with COPD can improve resting dyspnoea, fatigue^16^ and exercise tolerance.^19^ While paced walking to music tempo at 80% of the peak oxygen uptake (VO_2_ peak) may improve the exercise capacity for people with COPD,^16 19^ these studies used preselected music tempo/or songs played without commercial disruptions. Given the popularity of the radio among PR service users in this survey, this low-cost tool may present practical challenges when implementing music-based interventions, given that radio listening involves unpredictable disruptions (eg, adverts, speech). Furthermore, the songs played on the radio are often varied in tempos and potentially reduce the intervention effectiveness in maintaining a consistent exercise pace. It would be challenging to align the intensity of endurance walking to music tempo during the intervention while listening to the radio. This suggests the need for developing flexible interventions or platforms that can integrate spoken content and varied music tempo to replicate radio’s familiarity while providing a motivating listening experience.

### Potential barriers and safety concerns of exercising to music with headphones

Listening to music while wearing headphones when walking for an exercise outdoors raised safety concerns for our respondents including reducing the awareness of the surroundings and preventing communicating with other people. This is suggested to be the reason why the individual use of music is rare among pulmonary and cardiac rehabilitation programme attendees in Australia.^21^ In contrast, a previous pilot study of home-based paced walking to music programmes with and/or without headphones for people with COPD has been considered efficient without reporting any safety concerns.^19^ However, the proportion of those who used earbuds was not reported. In centre-based PR, the challenge of exercising to music using headphones may hinder staff communication and patient-clinicians’ interactions,^21^ but could be overcome by playing background music during the group exercise. During supervised aerobic exercise (eg, treadmill, bicycle) optional individual use of music using headphones could be advised. This would ensure the delivery of the instructions by the clinicians and individuals’ interaction during group activities and benefit from exercising to participants’ preferred music during the individual aerobic exercise. Reducing the surrounding awareness is the reported challenge with exercising to music using headphones at home by our respondents, which needs further exploration to successfully address that.

### Strengths and limitations

The survey was conducted at a single centre in the UK which may limit the generalisability of the findings to other settings or populations. However, given that respondents were recruited through the clinical service, the diversity of the respondents’ diagnoses, age and sex reflects the local and national PR population.^34^ The ethnicity of our respondents was not recorded, which might have implications for their responses regarding their music listening experience/behaviour. For example, the emotional response to music may differ from one ethnic group to another, which consequently may have an indirect impact on motivation to exercise. The importance of including the ethnicity when individualising music-based intervention is discussed elsewhere.^35^ The survey has identified several important areas for future implementation of music in PR service delivery. It would be beneficial to explore barriers, enablers and motivation for exercising to music in more detail through qualitative approaches.

## Conclusion

The findings suggest potential to integrate music as a tool to facilitate exercise adherence for people living with CRD attending PR. However, the diversity of music preferences, lack of experience and awareness of the potential benefits of exercising to music, and the identified concerns such as safety perceptions of outdoor walking with headphones in this population must be addressed for successful implementation. These findings may help future PR service delivery to implement music and/or develop a personalised music-based intervention to optimise exercise adherence, specifically when unsupervised in PR.

## Supplementary material

10.1136/bmjoq-2025-003666 online supplemental file 1

10.1136/bmjoq-2025-003666 online supplemental file 2

10.1136/bmjoq-2025-003666 online supplemental file 3

10.1136/bmjoq-2025-003666 online supplemental file 4

## Data Availability

All data relevant to the study are included in the article or uploaded as supplementary information.
