# Supplementary material for: Exploring music preferences, behaviours and experiences of exercising to music in pulmonary rehabilitation for individuals with chronic respiratory diseases: a cross-sectional survey
Source: BMJ Open Qual. 2026 Jan 12;15(1):e003666. doi: 10.1136/bmjoq-2025-003666 (PMC12815233; doi:10.1136/bmjoq-2025-003666)
Supplement: online supplemental file 1 [file bmjoq-15-1-s001.docx]

**The Use of Digital Health Technologies and Music in Daily Life and During Exercise: A service evaluation**

**Page 1: The Use of Digital Health Technologies and Music in**

**Daily Life and During Exercise**

We are interested in understanding how you use technology (e.g., Smartphones).

We are interested to know what type of music you prefer to listen to, when you listen and how you listen to music in your daily life and for exercise.

We are interested in your experiences and opinions to help us develop ways to help people living with Chronic Respiratory Diseases. There are no wrong answers.

Your responses are anonymous and confidential.

**This survey should take about 10 minutes to complete.**

**Page 2: Smartphone ownership and usage**

**(Smartphone is a mobile phone that performs many of the functions of a computer, typically having a touchscreen interface, internet access, and capable of running downloaded applications)**

1. **Do you currently own a Smartphone?**

Yes **If selected, skip to question 2.**

No

- 1. **Have you ever owned a Smartphone?**

Yes **If selected, skip to Page 3.**

No **If selected, skip to Page 4.**

1. **Who is your Smartphone manufacturer?**

| Apple (iPhone) | Google Pixel | Huawei |
| --- | --- | --- |
| Motorola | Nokia | Samsung |
| Sony | Xiaomi | Prefer not to say |
| Do not know | Other |  |

**If you selected Other, please specify your smartphone manufacturer:**

..................................................................................................

**Page 3: Smartphone ownership and usage**

1. **Do you know how to download a mobile application (app) onto a Smartphone?**

Yes

No

1. **Have you ever used a mobile application on a smartphone to monitor your health or exercise?**

Yes

No

1. **On a typical day, how much time do you typically spend using a Smartphone?**

Less than 1 hour per day

1-2 hours per day

2-3 hours per day

3-4 hours per day

4-7 hours per day

7-10 hours per day

More than 10 hours per day

**Page 4: Listening to music**

1. **How often do you listen to music (this could be in your home, in your car or out and about on any device)?**

Several times a day

Once a day

Several times a week

Several times a month

Once a month

Less often

I never listen to music

1. **How often, if at all, do you listen to any of the following?**

**[Please select from the table below] [If you selected other listening activities, please specify what listening activity in the box and select how often you listen to it]**

| **Listening activity** | **How often** | | | | | | |
| --- | --- | --- | --- | --- | --- | --- | --- |
|  | **Several times a day** | **Once a day** | **Several times a week** | **Several times a month** | **Once a month** | **Less often** | **Never** |
| 1. **Music Radio stations** |  |  |  |  |  |  |  |
| 1. **Personal music collection on NON-DIGITAL devices (CD, Vinyl, Cassette, etc.)** |  |  |  |  |  |  |  |
| 1. **Personal music on DIGITAL devices (Smartphone, Alexa, etc.)** |  |  |  |  |  |  |  |
| 1. **Music videos (e.g., YouTube)** |  |  |  |  |  |  |  |
| 1. **Speech-based radio stations (e.g., LBC, talkSPORT)** |  |  |  |  |  |  |  |
| 1. **Podcasts** |  |  |  |  |  |  |  |
| 1. **Audiobook** |  |  |  |  |  |  |  |
| 1. **Other listening activities, please specify:**   **……………………………………** |  |  |  |  |  |  |  |

1. **In which ways do you do the following listening activities?**

**[Please select from the table below] [You can select more than one way to do each listening activity]**

| **Listening activity** | **Ways of Listening** | | | | | |
| --- | --- | --- | --- | --- | --- | --- |
|  | **Mobile phone** | **Car Radio** | **TV radio channels Stereo/hi-fi/sound system (Alexa, CD, cassette)** | **PC/ Laptop** | **Other way of listening** | **Not applicable** |
| 1. **Music radio station** |  |  |  |  |  |  |
| 1. **Personal music collection** |  |  |  |  |  |  |
| 1. **Music videos (e.g., YouTube)** |  |  |  |  |  |  |
| 1. **Speech-based radio stations (e.g., LBC, talkSPORTS)** |  |  |  |  |  |  |
| 1. **Podcasts** |  |  |  |  |  |  |
| 1. **Audiobook** |  |  |  |  |  |  |

**If you select Other way of listening, please specify what listening way you do your listening activity:**

**…………………………………………..**

1. **Now think about the location. Where would you say you do the following listening activities?**

**[Please select from the table below] [You can select more than one location to do each listening activity]**

| **Listening activity** | **Where** | | | | | | |
| --- | --- | --- | --- | --- | --- | --- | --- |
|  | **In the home/ at home** | **In the Car/ Vehicle** | **In the gym** | **Outside while walking** | **Outside while doing other activities** | **Other location** | **Not applicable** |
| 1. **Music radio station** |  |  |  |  |  |  |  |
| 1. **Personal music collection on NON-DIGITAL devices (CD, Vinyl, Cassette, etc.)** |  |  |  |  |  |  |  |
| 1. **Personal music on DIGITAL devices (Smartphone, Alexa, etc.)** |  |  |  |  |  |  |  |
| 1. **Music videos (e.g., YouTube)** |  |  |  |  |  |  |  |
| 1. **Speech-based radio stations (e.g., LBC, talkSPORTS)** |  |  |  |  |  |  |  |
| 1. **Podcasts** |  |  |  |  |  |  |  |
| 1. **Audiobook** |  |  |  |  |  |  |  |

**If you selected Other location, please specify where you do your listening activity:**

**…………………………………………………**

1. **In general, what is your main reason for listening to the following listening activities?**

**[Please select from the table below] [You can select more than one reason for each listening activity]**

| **Listening activity** | **Reasons** | | | | | | |
| --- | --- | --- | --- | --- | --- | --- | --- |
|  | **For background listening** | **Finding it relaxing** | **Finding it interesting** | **To keep up with the latest developments** | **To learn something new** | **Other reason** | **Not applicable** |
| 1. **Music radio station** |  |  |  |  |  |  |  |
| 1. **Personal music collection** |  |  |  |  |  |  |  |
| 1. **Music videos (e.g., YouTube)** |  |  |  |  |  |  |  |
| 1. **Speech-based radio stations (e.g., LBC, talkSPORTS)** |  |  |  |  |  |  |  |
| 1. **Podcasts** |  |  |  |  |  |  |  |
| 1. **Audiobook** |  |  |  |  |  |  |  |

**If you select Other reason, please specify your reason for listening to the selected listening activity:**

**……………….…………….…………….**

1. **If you had to choose your top 3 favourite types of music, what would they be?**

**[Please select from the table below]**

| **Music Types** | **Top Favourite** | | |
| --- | --- | --- | --- |
|  | **Top one** | **Top two** | **Top three** |
| 1. **Alternative** |  |  |  |
| 1. **Classical** |  |  |  |
| 1. **Country** |  |  |  |
| 1. **Dubstep** |  |  |  |
| 1. **Hip-hop/ rap** |  |  |  |
| 1. **Instrumental** |  |  |  |
| 1. **Jazz** |  |  |  |
| 1. **K-pop** |  |  |  |
| 1. **Metal** |  |  |  |
| 1. **Motown** |  |  |  |
| 1. **Pop** |  |  |  |
| 1. **Reggae** |  |  |  |
| 1. **Rock & Roll** |  |  |  |
| 1. **No preference, I enjoy all types of music.** |  | | |
| 1. **I don’t really like any music.** |  | | |
| 1. **Other** |  | | |

**If you selected Other, please specify:**

……………………………………………………………………………………

1. **Where possible, name UP TO 5 of your favourite artists/bands.**
2. ………………………………
3. ………………………………
4. ………………………………
5. ………………………………
6. ………………………………
7. **Where possible, name UP TO 5 of your favourite songs.**
8. **………………………………**
9. **………………………………**
10. **………………………………**
11. **………………………………**
12. **………………………………**
13. **From what source do you listen to music?**

**[Please select all that apply]**

Amazon Music

Apple music/ i-Tunes

Deezer

Google Play music

I Heart Radio

Music channels on TV

Pandora

Radio

Sound Cloud

Spotify

YouTube

I don't listen to music

Other music websites or source

**If you selected Other music websites or sources, please specify from what websites or sources you listen to music:**

………...…………………………………………..

**
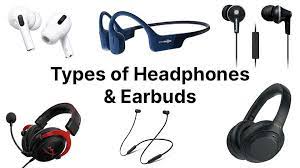
**

**(Headphones** are a term used for a device that features two ear pieces connected by a

headband and worn over the head**)**

**(Earbuds** are small devices that fit directly into the ear canal**)**

1. **Which of the following devices do you own for listening to music?**

**[Please select all that apply]**

| ☐ I don't own a device to listen to music **If selected, skip to question 16.** | | | |
| --- | --- | --- | --- |
| ☐ Alexa | ☐ Cassette Player | ☐ CD Player | ☐ iPad or iPhone or iPod (Apple) |
| **☐** Laptop or PC | **☐** MP3 Player | **☐** Radio | ☐ Smartphone or Tablet (not Apple) |
| ☐ Walkman | **☐** Other |  |  |

**If you selected Other, please specify what device you own to listen to music:**

......................................................

1. **How do you listen to music from the previously chosen devices?**

From the device’s own speakers (e.g., Alexa, iPad, etc.)

From wireless headphones and/or earbuds

From wired headphones and/or earbuds.

Other

**If you selected Other, please specify how you listen to music from the previously chosen devices:**

............................................

1. **What's your favourite thing about music?**

.....................................................................................

1. **Do you wear hearing aids?**

☐ Yes

☐ No

1. **In a typical week, how many days do you go for a walk outside? [e.g., walking to the shop/walking the dog]**

| 0 days/week | 1 days/week | 2 days/week | 3 days/week |
| --- | --- | --- | --- |
| 4 days/week | 5 days/week | 6 days/week | 7 days/week |

1. **In a typical week, how many days do you exercise?**

**[e.g., walking for exercise, exercise bike]**

| 0 days/week | 1 days/week | 2 days/week | 3 days/week |
| --- | --- | --- | --- |
| 4 days/week | 5 days/week | 6 days/week | 7 days/week |

1. **Which of the** **following have you ever listened to while exercising?**

**[Please select all that apply]**

I have never listened to anything while exercising

Audiobook

Music radio stations

Music videos

Online music service (Spotify, Amazon Music, etc.)

Personal music collection on NON-DIGITAL DEVICES (CD, Vinyl, Cassette)

Personal music on a DIGITAL DEVICE (Alexa, Smartphones, etc.)

Podcasts

Speech-based radio stations (e.g., LBC, talkSPORT radio)

Other

**If you selected Other, please specify what you have listened to while exercising:**

.....................................................................................

1. **If you were asked to listen to music through headphones whilst walking outside, Would have any of these concerns?**

**[Please select all that apply]**

No concerns

Distracts me from concentrating on my breathing

Headphones are not comfortable

Prevent me from communicating with other people

Reduce awareness of the surroundings

Other

**If you selected Other, please specify what concern you might likely to have:**

.....................................................................................

1. **If you were asked to listen to music through headphones whilst walking outside, What benefits might you expect to receive?**

**[Please select all that apply]**

No benefits

Helps to boost my mood

I focus on completing the walking exercise

I focus on maintaining my walking pace/rhythm

It reduces the sensation of breathlessness.

Other

**If you selected Other, please specify what benefits you might likely to receive:**

.....................................................................................

1. **Which of the following Chronic Respiratory Diseases have you been diagnosed with? [Please select all that apply]**

| Chronic Obstructive Pulmonary Disease (COPD) | Asthma |
| --- | --- |
| Interstitial Lung Disease (ILD) | Bronchiectasis |
| Other |  |

**If you selected Other, please specify what Chronic Respiratory Disease you have been diagnosed with:**

.....................................................................................

1. **Which age group do you belong to?**

| Less than 40 years | 40 - 49 years | 50 - 59 years |
| --- | --- | --- |
| 60 - 69 years | 70 - 79 years | 80 - 89 years |
| 90+ years |  |  |

1. **What is your gender?**

| Male | Female |
| --- | --- |
| Prefer Not to Say | Other |

**If you selected Other, please specify your gender:**

.....................................................................................

**That’s the end of the questions**

**Thank you for taking the time to complete this survey**
