# Supplementary material for: Exploring music preferences, behaviours and experiences of exercising to music in pulmonary rehabilitation for individuals with chronic respiratory diseases: a cross-sectional survey
Source: BMJ Open Qual. 2026 Jan 12;15(1):e003666. doi: 10.1136/bmjoq-2025-003666 (PMC12815233; doi:10.1136/bmjoq-2025-003666)
Supplement: online supplemental file 2 [file bmjoq-15-1-s002.docx]

Supplementary Table 3.A. Reported favourite artists, bands

| **No.** | **Reported favourite artists and bands** | **Frequency** |
| --- | --- | --- |
|  | Beatles | 11 |
|  | Queen | 11 |
|  | Bob Marley | 9 |
|  | ABBA | 8 |
|  | Rolling Stones | 7 |
|  | Dolly Parton | 6 |
|  | Elvis Presley | 6 |
|  | Meat Loaf | 6 |
|  | Pink Floyd | 5 |
|  | The Who | 5 |
|  | UB40 | 5 |
|  | Ed Sheeran | 4 |
|  | John Denver | 4 |
|  | Whitney Houston | 4 |
|  | Beach Boys | 3 |
|  | David Bowie | 3 |
|  | Eagles | 3 |
|  | Electric Light Orchestra | 3 |
|  | Elton John | 3 |
|  | Frank Sinatra | 3 |
|  | Johnny Cash | 3 |
|  | Michael Ball | 3 |
|  | Nat King Cole | 3 |
|  | Shirley Bassey | 3 |
|  | Simply Red | 3 |
|  | Take That | 3 |
|  | AC/DC | 2 |
|  | Bee Gees | 2 |
|  | Billy Fury | 2 |
|  | Billy Joel | 2 |
|  | Buddy Holly | 2 |
|  | Celine Dion | 2 |
|  | Cliff Richard | 2 |
|  | Coldplay | 2 |
|  | Dire Straits | 2 |
|  | Dr. Hook & the Medicine Show | 2 |
|  | Engelbert Humperdinck | 2 |
|  | Everly Brothers | 2 |
|  | Jim Reeves | 2 |
|  | Neil Diamond | 2 |
|  | Rod Stewart | 2 |
|  | Roy Orbison | 2 |
|  | Russell Watson | 2 |
|  | Simon & Garfunkel | 2 |
|  | Stylistics | 2 |
|  | A. R. Rahman | 1 |
|  | Adele | 1 |
|  | Alfie Boe | 1 |
|  | André Rieu | 1 |
|  | Andrea Bocelli | 1 |
|  | Andy Williams | 1 |
|  | Anirudh Ravichander | 1 |
|  | Asha Bhosle | 1 |
|  | Big Bopper | 1 |
|  | Black Sabbath | 1 |
|  | Blue Öyster Cult | 1 |
|  | Blur | 1 |
|  | Bob Dylan | 1 |
|  | Bon Jovi | 1 |
|  | Boney M. | 1 |
|  | Boz Scaggs | 1 |
|  | Bryn Terfel | 1 |
|  | Carpenters | 1 |
|  | Celtic Worship | 1 |
|  | Charles Pride | 1 |
|  | Chilcott | 1 |
|  | Chris Rea | 1 |
|  | City of Birmingham Symphony Orchestra (Orchestra) | 1 |
|  | David Essex | 1 |
|  | Debbie Harry | 1 |
|  | Deep Purple | 1 |
|  | Def Leppard | 1 |
|  | Diana ross | 1 |
|  | DIDO | 1 |
|  | Dionne Warwick | 1 |
|  | Dobie Gray | 1 |
|  | Donald Lawrence and the Tri-City Singers | 1 |
|  | Doris Day | 1 |
|  | Drifters | 1 |
|  | Duran Duran | 1 |
|  | Dusty Springfield | 1 |
|  | Edward Elgar | 1 |
|  | Elaine Paige | 1 |
|  | Elevation Worship | 1 |
|  | Ella Fitzgerald | 1 |
|  | ELO | 1 |
|  | Emmylou Harris | 1 |
|  | Errol Brown | 1 |
|  | Eva Cassidy | 1 |
|  | Fauré | 1 |
|  | Fleetwood Mac | 1 |
|  | Four Seasons band | 1 |
|  | Four Tops | 1 |
|  | Ginger Baker | 1 |
|  | Gladys Knight & the Pips | 1 |
|  | Goodall | 1 |
|  | Guns N' Roses | 1 |
|  | Harry Connick Jr. | 1 |
|  | Herman's Hermits | 1 |
|  | Hezekiah Walker | 1 |
|  | Hot Chocolate | 1 |
|  | Imagine Dragons | 1 |
|  | Jack white | 1 |
|  | Jimi Hendrix | 1 |
|  | Jimmy Nail | 1 |
|  | Joan Sutherland | 1 |
|  | John Hogan | 1 |
|  | John Lennon | 1 |
|  | John Prine | 1 |
|  | Josh Groban | 1 |
|  | Julie Andrews | 1 |
|  | Julie London | 1 |
|  | K. J. Yesudas | 1 |
|  | Katherine Jenkins | 1 |
|  | Katrina And The Waves | 1 |
|  | Ken Booth | 1 |
|  | Killers | 1 |
|  | Kinks | 1 |
|  | Kishore Kumar | 1 |
|  | Lata Mangeshkar | 1 |
|  | Led Zeppelin | 1 |
|  | Leonid and Friends | 1 |
|  | Lionel Richie | 1 |
|  | Lloyd-Webber | 1 |
|  | M People | 1 |
|  | M. Jayachandran | 1 |
|  | Madness | 1 |
|  | Marvin Gaye | 1 |
|  | Matt Monro | 1 |
|  | Max Bruch | 1 |
|  | Meatloaf | 1 |
|  | Michael Jackson | 1 |
|  | Michael W. Smith | 1 |
|  | Miles Davis | 1 |
|  | Mozart | 1 |
|  | Nick Cave | 1 |
|  | Nina Simone | 1 |
|  | Oasis | 1 |
|  | Orchestral Manoeuvres in the Dark | 1 |
|  | Ozzy Osbourne | 1 |
|  | Pat O'Neill | 1 |
|  | Pet Shop Boys | 1 |
|  | Phil Collins | 1 |
|  | PJ Harvey | 1 |
|  | Prince | 1 |
|  | Pulp | 1 |
|  | Raffi | 1 |
|  | Richard Smallwood and Vision | 1 |
|  | Rory Gallagher | 1 |
|  | Roxy Music | 1 |
|  | Ry Cooder | 1 |
|  | Sabri brothers | 1 |
|  | Sam the Sham | 1 |
|  | Sex Pistols | 1 |
|  | Shakira | 1 |
|  | Showaddywaddy | 1 |
|  | Sinach | 1 |
|  | Siouxsie Sioux | 1 |
|  | Slade | 1 |
|  | Small Faces | 1 |
|  | Status Quo | 1 |
|  | Steeleye Span | 1 |
|  | Stormzy | 1 |
|  | Style Council | 1 |
|  | Sweet | 1 |
|  | T. Rex | 1 |
|  | Temptations | 1 |
|  | The Band | 1 |
|  | The Cure | 1 |
|  | The Drifters | 1 |
|  | The KLF | 1 |
|  | The Police | 1 |
|  | The Shadows | 1 |
|  | The Smiths | 1 |
|  | Tina Turner | 1 |
|  | Tod Dulaney | 1 |
|  | Tom Jones | 1 |
|  | Tom Waits | 1 |
|  | Traveling Wilburys | 1 |
|  | Wet Wet Wet | 1 |
|  | Whitesnake | 1 |

Supplementary Table 3.B. Reported favourite songs

| **No.** | **Reported favourite songs** | **Frequency** |
| --- | --- | --- |
|  | Bohemian Rhapsody | 6 |
|  | Red Red Wine | 6 |
|  | I Will Always Love You | 3 |
|  | No Woman No Cry | 3 |
|  | I Want to Break Free | 3 |
|  | Bat Out of Hell | 2 |
|  | Bridge Over Troubled Water | 2 |
|  | Chain Reaction | 2 |
|  | Cold Heart | 2 |
|  | Common People | 2 |
|  | Livin' on a Prayer | 2 |
|  | Penny Lane | 2 |
|  | Pretty Woman | 2 |
|  | She Loves You | 2 |
|  | Stand by Your Man | 2 |
|  | Waterloo | 2 |
|  | All Night Long | 2 |
|  | Bang Bang | 2 |
|  | (I Can't Get No) Satisfaction | 1 |
|  | 3 steps to heaven | 1 |
|  | A Forest | 1 |
|  | A Sky full of Stars | 1 |
|  | Albatross | 1 |
|  | All I Ask of You | 1 |
|  | All I Have to Do is Dream | 1 |
|  | Anarchy in the U.K | 1 |
|  | Baby Love | 1 |
|  | Baby Please Don't Go | 1 |
|  | Back Street Girl | 1 |
|  | Badru Alaina | 1 |
|  | Because He Lives | 1 |
|  | Between a Man and a Woman | 1 |
|  | Blue Skies | 1 |
|  | Blue Sky | 1 |
|  | Blue Suede Shoes | 1 |
|  | Boardwalk | 1 |
|  | Break Away | 1 |
|  | Cancelling Out | 1 |
|  | Can't Help Falling in Love | 1 |
|  | Carnival Is Over | 1 |
|  | Changes | 1 |
|  | Chantilly Lace | 1 |
|  | Children of the Grave | 1 |
|  | Clarinet Concerto Mozart Adagio | 1 |
|  | C'mon You Know | 1 |
|  | Coat of Many Colours | 1 |
|  | Comfortably Numb | 1 |
|  | Company | 1 |
|  | Cracklin' Rosie | 1 |
|  | Cry Me a River | 1 |
|  | Dance With My Father | 1 |
|  | Dancing Queen | 1 |
|  | Daniel | 1 |
|  | Dead Ringer for Love | 1 |
|  | Desperado | 1 |
|  | Do The Strand | 1 |
|  | Do You Want to Know a Secret | 1 |
|  | Dolce Suono from Lucia di Lammermoor | 1 |
|  | Don't Fear the Reaper | 1 |
|  | Don't Stop Me Now | 1 |
|  | Doubt | 1 |
|  | Down by the Water | 1 |
|  | Drift Away | 1 |
|  | Driving Home for Christmas | 1 |
|  | Ebony Eyes | 1 |
|  | Echoes | 1 |
|  | El Taxi | 1 |
|  | Eloise | 1 |
|  | Enola Gay | 1 |
|  | Everything I Own | 1 |
|  | Falling | 1 |
|  | Fernando | 1 |
|  | Fly Me to the Moon | 1 |
|  | Gimme Shelter | 1 |
|  | God Only Knows | 1 |
|  | Goldfinger | 1 |
|  | Got You Under My Skin | 1 |
|  | Halfway to Paradise | 1 |
|  | Hard Day's Night | 1 |
|  | Hell Ain't a Bad Place to Be | 1 |
|  | Hello Again | 1 |
|  | Hello Hello | 1 |
|  | Help | 1 |
|  | Highway to Hell | 1 |
|  | Hips Don't Lie | 1 |
|  | Holding Back the Years | 1 |
|  | Hotel California | 1 |
|  | House for Kings | 1 |
|  | House of Fun | 1 |
|  | How Soon Is Now | 1 |
|  | I Dreamed a Dream | 1 |
|  | I Drove All Night | 1 |
|  | I Heard It Through the Grapevine | 1 |
|  | I Want to Hold Your Hand | 1 |
|  | If You Don't Know Me | 1 |
|  | I'll Be There | 1 |
|  | I'm Alive | 1 |
|  | I'm Not in Love | 1 |
|  | Imagine | 1 |
|  | In the Ghetto | 1 |
|  | Is This Love | 1 |
|  | It Doesn't Matter Anymore | 1 |
|  | It Is Not Unusual | 1 |
|  | It's All Over Now | 1 |
|  | It's Easy to Be in Love with You | 1 |
|  | Jireh | 1 |
|  | Jolene | 1 |
|  | King of the Road | 1 |
|  | Leaves of Gold | 1 |
|  | Life on Mars | 1 |
|  | Lily Was Here | 1 |
|  | Long Way from Home | 1 |
|  | Love Changes Everything | 1 |
|  | Love Is All Around | 1 |
|  | Major Tom | 1 |
|  | Memory | 1 |
|  | Mo Mo Land | 1 |
|  | Money for Nothing | 1 |
|  | Money, Money, Money | 1 |
|  | Moonlight Sonata | 1 |
|  | My Love and Devotion | 1 |
|  | My Way | 1 |
|  | New York, New York | 1 |
|  | Night Fever | 1 |
|  | O Holy Night | 1 |
|  | One Love | 1 |
|  | One Night with You | 1 |
|  | Ong Ong | 1 |
|  | Paradise by the Dashboard Light | 1 |
|  | Peggy Sue | 1 |
|  | Pinball Wizard | 1 |
|  | Purple Rain | 1 |
|  | Radio Ga Ga | 1 |
|  | Red Right Hand | 1 |
|  | Return to Sender | 1 |
|  | Revelation Song | 1 |
|  | Ride of the Valkyries | 1 |
|  | Ring of Fire | 1 |
|  | Rumble | 1 |
|  | Run to You | 1 |
|  | Secret Love | 1 |
|  | Seven Nation | 1 |
|  | Stairway to Heaven | 1 |
|  | Stand by Me | 1 |
|  | Start Me Up | 1 |
|  | Strawberry Fields | 1 |
|  | Sufficient for Today | 1 |
|  | Sugar Sugar | 1 |
|  | Sunshine Girl | 1 |
|  | Sweet Dreams | 1 |
|  | Tender | 1 |
|  | That'll Be the Day | 1 |
|  | That's What Friends Are For | 1 |
|  | The Joker and the Queen | 1 |
|  | The Lady in Red | 1 |
|  | The Last Walk | 1 |
|  | The Paris Match | 1 |
|  | The Reflex | 1 |
|  | The Universal | 1 |
|  | The Way We Were | 1 |
|  | The Weight | 1 |
|  | Three Little Birds | 1 |
|  | Thunderstruck | 1 |
|  | Time | 1 |
|  | West End Girls | 1 |
|  | Whatever | 1 |
|  | When I Fall in Love | 1 |
|  | When the Child Is Born | 1 |
|  | Whenever I See Your Face | 1 |
|  | White Wedding | 1 |
|  | Who Wants to Live Forever | 1 |
|  | Windmills of Your Mind | 1 |
|  | Wonderful World | 1 |
|  | Wooden Heart | 1 |
|  | Wooly Bully | 1 |
|  | Wreck of the Old 97 | 1 |
|  | Writing My Own Queen Song | 1 |
|  | Ya Nabi | 1 |
|  | You Make My Pants Want to Get Up and Dance | 1 |
|  | You Wanna Hold My Hand | 1 |
|  | You Will Never Walk Alone | 1 |
|  | Another One Bites the Dust | 1 |
|  | Bless Me (Prayer of Jabez) | 1 |
|  | Brahms' Requiem | 1 |
|  | Cabaret | 1 |
|  | Emily | 1 |
|  | Eternal Light | 1 |
|  | Every Praise | 1 |
|  | Fauré's Requiem | 1 |
|  | Gloria | 1 |
|  | Hero | 1 |
|  | Love Games | 1 |
|  | Mona Lisa | 1 |
|  | No Weapon | 1 |
|  | Rejoice | 1 |
|  | Sisters and Brothers | 1 |
|  | Songs from Cats | 1 |
|  | West Side Story | 1 |
|  | Your Love Divine | 1 |
