# Supplementary material for: Exploring music preferences, behaviours and experiences of exercising to music in pulmonary rehabilitation for individuals with chronic respiratory diseases: a cross-sectional survey
Source: BMJ Open Qual. 2026 Jan 12;15(1):e003666. doi: 10.1136/bmjoq-2025-003666 (PMC12815233; doi:10.1136/bmjoq-2025-003666)
Supplement: online supplemental file 3 [file bmjoq-15-1-s003.DOC]

Supplementary Table 2A. Favourite genre reported by respondents

| **No.** | **Favourite Genre** | **Frequency, n (%)** |
| --- | --- | --- |
|  | Pop | 39 (36%) |
|  | Country | 38 (35%) |
|  | Motown | 31 (28%) |
|  | Classical | 31 (28%) |
|  | Reggae | 25 (23%) |
|  | Rock & Roll | 21 (19%) |
|  | Instrumental | 15 (14%) |
|  | Alternative | 11 (10%) |
|  | Jazz | 7 (6%) |
|  | Metal | 6 (5%) |
|  | Hip-hop/rap | 3 (3%) |
|  | K-pop | 2 (2%) |

Supplementary Table 2B. Parings of the genres were frequently chosen together by respondents

| **No.** | **Genre 1** | **Genre 2** | **Frequency** |
| --- | --- | --- | --- |
|  | Alternative | Classical | 4 |
|  | Alternative | Country | 1 |
|  | Alternative | Instrumental | 2 |
|  | Alternative | Jazz | 2 |
|  | Alternative | K-pop | 1 |
|  | Alternative | Metal | 2 |
|  | Alternative | Motown | 3 |
|  | Alternative | Pop | 2 |
|  | Alternative | Reggae | 5 |
|  | Alternative | Rock & Roll | 2 |
|  | Classical | Country | 13 |
|  | Classical | Instrumental | 9 |
|  | Classical | Jazz | 4 |
|  | Classical | Metal | 1 |
|  | Classical | Motown | 6 |
|  | Classical | Pop | 10 |
|  | Classical | Reggae | 4 |
|  | Classical | Rock & Roll | 4 |
|  | Country | Instrumental | 3 |
|  | Country | Jazz | 3 |
|  | Country | Metal | 1 |
|  | Country | Motown | 10 |
|  | Country | Pop | 17 |
|  | Country | Reggae | 8 |
|  | Country | Rock & Roll | 8 |
|  | Hip-hop/rap | Pop | 1 |
|  | Hip-hop/rap | Reggae | 3 |
|  | Instrumental | Jazz | 1 |
|  | Instrumental | K-pop | 1 |
|  | Instrumental | Metal | 1 |
|  | Instrumental | Motown | 3 |
|  | Instrumental | Pop | 6 |
|  | Instrumental | Reggae | 2 |
|  | Instrumental | Rock & Roll | 1 |
|  | Jazz | K-pop | 1 |
|  | Jazz | Metal | 1 |
|  | Jazz | Motown | 2 |
|  | Jazz | Pop | 2 |
|  | Jazz | Rock & Roll | 1 |
|  | K-pop | Metal | 2 |
|  | K-pop | Motown | 1 |
|  | K-pop | Pop | 1 |
|  | Metal | Motown | 2 |
|  | Metal | Pop | 3 |
|  | Metal | Reggae | 1 |
|  | Motown | Pop | 13 |
|  | Motown | Reggae | 8 |
|  | Motown | Rock & Roll | 8 |
|  | Pop | Reggae | 11 |
|  | Pop | Rock & Roll | 9 |
|  | Reggae | Rock & Roll | 6 |
