# Supplementary material for: Exploring music preferences, behaviours and experiences of exercising to music in pulmonary rehabilitation for individuals with chronic respiratory diseases: a cross-sectional survey
Source: BMJ Open Qual. 2026 Jan 12;15(1):e003666. doi: 10.1136/bmjoq-2025-003666 (PMC12815233; doi:10.1136/bmjoq-2025-003666)
Supplement: online supplemental file 4 [file bmjoq-15-1-s004.DOC]

Supplementary Table 1. Frequency, location and reasons for listening to different listening activities

|  | **Music radio station** | | | | **Personal music collection on non-digital devices (CD, Vinyl, Cassette, etc.)** | | **Personal music on digital devices (Smartphone, Alexa, etc.)** | **Music videos (e.g., YouTube)** | | | | **Speech-based radio stations (e.g., LBC, talkSPORTS)** | | **Podcasts** | | | **Audiobook** | | |  |  |
| --- | --- | --- | --- | --- | --- | --- | --- | --- | --- | --- | --- | --- | --- | --- | --- | --- | --- | --- | --- | --- | --- |
| **How often** | **Participants, n (%)** | | | | | | | | | | | | | | | | | | |  |  |
| Several times a day | 29 (27) | | | | 4 (4) | | 14 (13) | 4 (4) | | | | 11 (10) | | 3 (3) | | | 2 (2) | | |  |  |
| Once a day | 13 (12) | | | | 5 (5) | | 5 (5) | 3 (3) | | | | 3 (3) | | 1 (1) | | | 0 (0) | | |  |  |
| Several times a week | 17 (16) | | | | 13 (12) | | 15 (14) | 16 (15) | | | | 9 (8) | | 4 (4) | | | 5 (5) | | |  |  |
| Several times a month | 11 (10) | | | | 7 (6) | | 6 (5) | 9 (8) | | | | 7 (6) | | 4 (4) | | | 1 (1) | | |  |  |
| Once a month | 1 (1) | | | | 2 (2) | | 4 (4) | 4 (4) | | | | 0 (0) | | 1 (1) | | | 1 (1) | | |  |  |
| Less often | 12 (11) | | | | 24 (22) | | 13 (12) | 13 (12) | | | | 11 (10) | | 8 (7) | | | 7 (6) | | |  |  |
| Never | 22 (20) | | | | 48 (44) | | 45 (41) | 54 (49) | | | | 62 (57) | | 80 (73) | | | 85 (78) | | |  |  |
| **Where** | **Participants, n (%)** | | | | | | | | | | | | | | | | | | |  |  |
| In the car/ Vehicle | 73 (67) | | | | 16 (15) | | 14 (13) | 2 (2) | | | | 21 (19) | | 5 (5) | | | 5 (5) | | |  |  |
| In the home/ at home | 68 (62) | | | | 46 (42) | | 38 (35) | 41 (38) | | | | 27 (25) | | 15 (14) | | | 13 (12) | | |  |  |
| Outside while walking | 4 (4) | | | | 5 (5) | | 5 (5) | 2 (2) | | | | 2 (2) | | 1 (1) | | | 3 (3) | | |  |  |
| Outside while doing other activities | 11 (10) | | | | 5 (5) | | 8 (7) | 2 (2) | | | | 1 (1) | | 0 (0) | | | 0 (0) | | |  |  |
| In the gym | 6 (5) | | | | 1 (1) | | 5 (5) | 1 (1) | | | | 0 (0) | | 1 (1) | | | 0 (0) | | |  |  |
| Other location | 2 (2) | | | | 0 (0) | | 2 (2) | 4 (4) | | | | 0 (0) | | 2 (2) | | | 0 (0) | | |  |  |
| No applicable | 20 (18) | | | | 55 (50) | | 67 (61) | 66 (60) | | | | 72 (66) | | 93 (85) | | | 96 (88) | | |  |  |
| **Why** | **Participants, n (%)** | | | | | | | | | | | | | | | | | | |  |  |
| For background listening | | 62 (57) | | | | 22 (20) | | | 7 (6) | | 13 (12) | | | | 3 (3) | | | 1 (1) | |  |  |
| Finding it relaxing | | 51 (47) | | | | 51 (47) | | | 23 (21) | | 5 (5) | | | | 2 (2) | | | 7 (6) | |  |  |
| Finding it interesting | | 26 (24) | | | | 14 (13) | | | 18 (16) | | 22 (20) | | | | 12 (11) | | | 8 (7) | |  |  |
| To keep up with the latest developments | | 8 (7) | | | | 1 (1) | | | 8 (7) | | 19 (17) | | | | 7 (6) | | | 3 (3) | |  |  |
| To learn something new | | 6 (5) | | | | 2 (2) | | | 9 (8) | | 15 (14) | | | | 8 (7) | | | 5 (5) | |  |  |
| Other reason | | 2 (2) | | | | 5 (5) | | | 2 (2) | | 3 (3) | | | | 1 (1) | | | 1 (1) | |  |  |
| Not applicable | | 21 (19) | | | | 47 (43) | | | 69 (63) | | 75 (69) | | | | 93 (85) | | | 96 (88) | |  |  |
| **Ways of Listening** | | **Participants, n (%)** | | | | | | | | | | | | | | | | | |  |  |
| Smartphone | | | | 14 (13) | 18 (16) | | | | | | 18 (16) | | | 7 (6) | | | 8 (7) | | | 7 (6) | |
| Car Radio | | | | 63 (58) | 10 (9) | | | | | | 2 (2) | | | 17 (16) | | | 1 (1) | | | 2 (2) | |
| TV radio channels/stereo/hi-fi/sound system (Alexa, CD, cassette) | | | | 47 (43) | 30 (27) | | | | | | 19 (17) | | | 22 (20) | | | 4 (4) | | | 2 (2) | |
| PC/Laptop | | | | 6 (5) | 12 (11) | | | | | | 16 (15) | | | 5 (5) | | | 5 (5) | | | 4 (4) | |
| Other way of listening | | | | 6 (5) | 9 (8) | | | | | | 2 (2) | | | 2 (2) | | | 2 (2) | | | 0 (0) | |
| Not applicable | | | | 19 (17) | 55 (50) | | | | | | 66 (60) | | | 72 (66) | | | 94 (86) | | | 98 (90) | |

Abbreviations: CD = Compact Disc; PC = Personal Computer; LBC = Leading Britain’s Conversation (UK talk radio station)
